# Perspective on Theoretical and Experimental Advances in Atmospheric Photochemistry

**DOI:** 10.1021/acs.jpca.4c03481

**Published:** 2024-07-18

**Authors:** Basile F. E. Curchod, Andrew J. Orr-Ewing

**Affiliations:** School of Chemistry, University of Bristol, Bristol BS8 1TS, U.K.

## Abstract

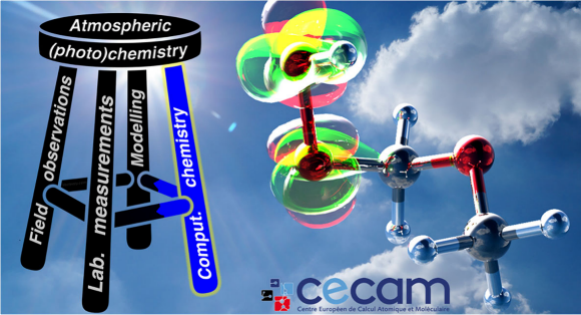

Research that explores the chemistry of Earth’s atmosphere is central to the current understanding of global challenges such as climate change, stratospheric ozone depletion, and poor air quality in urban areas. This research is a synergistic combination of three established domains: earth observation, for example, using satellites, and *in situ* field measurements; computer modeling of the atmosphere and its chemistry; and laboratory measurements of the properties and reactivity of gas-phase molecules and aerosol particles. The complexity of the interconnected chemical and photochemical reactions which determine the composition of the atmosphere challenges the capacity of laboratory studies to provide the spectroscopic, photochemical, and kinetic data required for computer models. Here, we consider whether predictions from computational chemistry using modern electronic structure theory and nonadiabatic dynamics simulations are becoming sufficiently accurate to supplement quantitative laboratory data for wavelength-dependent absorption cross-sections, photochemical quantum yields, and reaction rate coefficients. Drawing on presentations and discussions from the CECAM workshop on *Theoretical and Experimental Advances in Atmospheric Photochemistry* held in March 2024, we describe key concepts in the theory of photochemistry, survey the state-of-the-art in computational photochemistry methods, and compare their capabilities with modern experimental laboratory techniques. From such considerations, we offer a perspective on the scope of computational (photo)chemistry methods based on rigorous electronic structure theory to become a fourth core domain of research in atmospheric chemistry.

## 1 Introduction

Beyond its most abundant constituent gases N_2_, O_2_, Ar, and CO_2_, the chemical composition of the Earth’s lower atmosphere is a complicated mixture of volatile organic compounds (VOCs), oxygen containing gases (e.g., ozone, nitrogen oxides, SO_2_, and inorganic acids), free radicals, and aerosol particles.^1−3^ In the upper atmosphere, the chemistry is also influenced by metals ablated from meteors.^4^ Although many of these minor constituents are present only at trace levels of parts per million (ppm), parts per billion (ppb), or lower, they have a profound impact on the composition and properties of the atmosphere. The chemical complexity of the troposphere and stratosphere is a consequence of various natural and anthropogenic emissions from the planet’s land and ocean surfaces, together with a rich system of photochemical oxidation reactions driven by the energy in sunlight. Current understanding of the physical properties of the atmosphere, its meteorology, and the sources, transport, reactions, and losses of its chemical components can explain numerous phenomena, such as the ozone layer in the stratosphere; the catalytic ozone destruction cycles that lead to the annual Antarctic ozone hole; increases in the abundance of greenhouse gases in the troposphere; the impact of human activity on climate; and poor air quality in urban areas arising from VOC and NO_*x*_ (NO and NO_2_) emissions.^1−3^ In many cases, this understanding is now quantitative, albeit with uncertainties that arise from remaining gaps in our mechanistic understanding or from incomplete data for anthropogenic and natural emissions budgets.^5−7^

The active areas of research that have contributed so effectively to our current understanding of the composition of the atmosphere can broadly be divided into three categories: atmospheric measurements, laboratory studies, and computer modeling. Observational measurements of atmospheric composition use analytical instruments mounted on satellite, aircraft, balloon, ship, or ground-based platforms. They might apply spectroscopic, chromatographic, and mass spectrometric techniques to identify and quantify different chemical species, including VOCs or reactive free radicals (for example, OH, NO_3_ and halogen monoxides). Converting *in situ* spectroscopic measurements into information about chemical composition requires access to quantitative absorption spectra of individual molecules and radicals, for which databases such as HITRAN,^8^ the MPI-Mainz UV/vis Spectral Atlas,^9^ and NASA/JPL^10^ and IUPAC^11^ expert evaluations are convenient, curated compilations. The range of available measurement platforms provides compositional data on global, regional, and local scales, and collaborative field campaigns will often include a suite of instruments that can provide complementary data for a range of chemical compounds.^12^

The interpretation of the information gathered from field measurements requires computer models that incorporate treatments of the likely sources, sinks, transport, and photochemical reactions of numerous chemical constituents.^2^ At the heart of these computer models, detailed reaction mechanisms describe the atmospheric chemistry of interest.^13,14^ For quantitative predictions of atmospheric lifetimes and reaction pathways, these mechanisms must include laboratory data derived from measurements by physical chemists of absorption spectra, photochemical quantum yields, reaction rate coefficients, and reaction product branching ratios.^15,16^ If heterogeneous (multiphase) chemistry is to be incorporated in the models, for example to account for uptake and chemical processing of gaseous species by aerosol particles, laboratory data are also needed for vapor pressures, surface tensions, uptake coefficients, solubilities and supersaturation, viscosities, and reaction rates in condensed media.^17,18^ Continuous feedback between the three pillars of field observation,^1,2,19^ computer modeling,^2,20^ and laboratory measurements^21^ deepens our understanding of atmospheric chemistry, and it reveals gaps in our current knowledge that stimulate further advances in the discipline. These advances are vital because a robust understanding of atmospheric chemistry informs the expert reports and advice supplied to national governments or international bodies making decisions about environmental policy.^22−24^

Nevertheless, the sheer complexity of much of the chemistry of the atmosphere presents challenges to computer modelers and to laboratory-based physical chemists. Incorporation of comprehensive chemical schemes into computer models of atmospheric chemistry can make them prohibitively expensive to run, so reduced chemical schemes are often preferred.^25^ Meanwhile, the number of possible photochemical processes and chemical reactions that need to be studied experimentally is beyond the capacity of research laboratories worldwide.^26,27^ Laboratory measurements should determine not just reaction rate coefficients (*k*) or wavelength-dependent absorption cross-sections (σ(λ)) and photochemical quantum yields (Φ(λ)), but also their dependence on pressure and temperature.^15^ To illustrate the challenge, the Master Chemical Mechanism (MCM v3.3.1) currently includes 5832 species and 17224 reactions.^28^ Established strategies to address this complexity are exemplified by the development of structure–activity relationships (SARs),^27^ and mechanism reduction techniques such as the Common Reactive Intermediate (CRI) approach.^29−31^ Even so, the reduced description of the oxidation reactions of isoprene (a terpene emitted in large quantities by trees and plants^6^) with OH, NO_3_, and ozone in the CRI v2.2 model includes 186 reaction steps and 56 reactant species,^25^ which is an order of magnitude reduced from MCM v3.3.1.

A CECAM Flagship Workshop *Theoretical and Experimental Advances in Atmospheric Photochemistry* held in Lausanne, Switzerland, in March 2024 brought together early career and established theoretical and physical chemists with common interests in atmospheric chemistry to review the role that advanced computational (photo)chemistry methods can now play to help resolve this complexity bottleneck. Drawing on the outcomes of this workshop, we argue here that computational chemistry research applying state-of-the-art methods from quantum chemistry, chemical dynamics simulations, machine learning, and perhaps the developing capabilities in quantum computing can offer an increasingly robust alternative to the painstaking and time-consuming laboratory measurements of photochemical pathways that rely on specialist and expensive equipment found only in a limited number of laboratories worldwide. If predictions from modern computational chemistry of quantities such σ(λ,*T*,*p*), Φ(λ,*T*,*p*), and *k*(*T*,*p*) can approach the accuracy of laboratory studies, then with widening access to suitable codes and computational hardware, the capacity for valuable data generation could outstrip laboratory measurements. In the words of workshop participant Professor Joseph Francisco (University of Pennsylvania), at this stage of maturity, computational chemistry offers a “fourth pillar” for atmospheric chemistry research. Such maturity may already be at hand for calculation of reactions in their ground electronic states,^32^ but description of excited-state photochemistry remains at the cutting-edge of modern theoretical developments.

In this Perspective, we provide an overview of the current state-of-the-art of theoretical and computational research in atmospheric photochemistry as presented at the CECAM workshop, compare it to current experimental capabilities, and propose future directions to consolidate computational chemistry as a central pillar of atmospheric chemistry research. Our focus is on photochemical processes, which present particular challenges to theoretical and computational chemistry and are perhaps under-represented in current atmospheric chemistry models but are central to the chemistry of the troposphere and stratosphere.

Filtering of the flux of solar ultraviolet radiation by molecules present at higher altitudes regulates the photochemistry that occurs in the stratosphere and the troposphere. Photochemical rate coefficients, *J*, depend on the solar zenith angle (θ) and wavelength-dependent flux, *F*(θ,λ):^1−3^
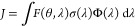
1

Here the integral is over the solar spectrum of wavelengths and gives rate coefficients that depend on altitude through the behavior of *F*(θ,λ). Wavelengths shorter than about 200 nm in the downwelling solar flux are effectively absorbed by O_2_ at high altitudes, and only wavelengths longer than ∼290 nm penetrate the stratospheric ozone layer with sufficient flux to drive photochemistry in the troposphere.^33^ Consequently, tropospheric photochemistry is dominated by molecules such as ozone, nitrogen dioxide (NO_2_), nitrous acid (HONO), and various classes of VOCs such as carbonyl compounds with near-UV chromophores. For VOC photochemistry to have a significant impact on tropospheric composition, the solar photolysis rates must compete with the rates of other removal processes such as the reaction of the VOC with OH radicals. The shorter UV wavelengths in the stratosphere can photochemically cleave C–Cl bonds, leading to the release of ozone-depleting Cl atoms from chlorofluorocarbons (CFCs) that are photochemically inactive in the troposphere. Such constraints narrow the focus of computational atmospheric photochemistry research but still leave a wide range of problems to tackle.

**Figure 1:**
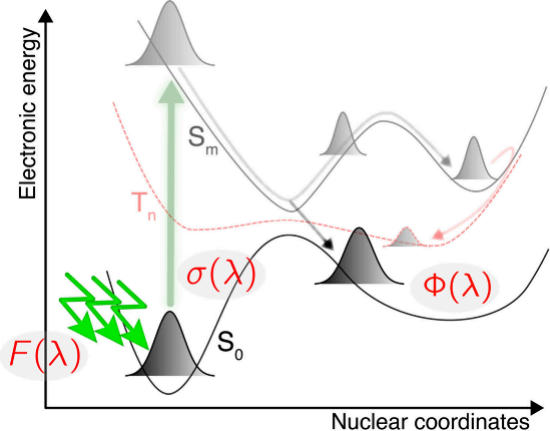
A schematic diagram illustrating key processes in molecular photochemistry initiated by absorption of the solar flux of UV and visible light (*F*(λ)). Absorption is represented by the green vertical arrow and is to an excited electronic state typically of the same electronic spin (here, singlet states S_0_, S_*m*_). The strength of absorption is quantified by the wavelength-dependent absorption cross-section, σ(λ). Subsequent dynamics on excited and ground electronic states (solid and dashed lines) determine branching between photochemical pathways such as triplet state (T_*n*_) population and formation of photoproducts, as illustrated by nonadiabatic evolution of the nuclear wave functions (gray) describing the molecular structures. Quantum yields Φ(λ) quantify this branching.

Here, we discuss the atmospheric chemistry questions that laboratory experiments and computational chemistry can address, the best approaches currently available, and the priorities for future developments. Central to this discussion, we emphasize that rigorous intercomparison between theoretical predictions and experimental laboratory measurements is necessary to guide the field of computational atmospheric photochemistry to maturity. We first break down conceptually a photochemical reaction into a sequence of steps, the study of each of which requires different experimental or computational strategies.^34^ These steps are illustrated schematically in Figure 1. The first is the absorption of a photon of ultraviolet (UV) or visible light of sufficient energy to promote a molecule (the chromophore) to an electronically excited state in which one or more valence electrons occupy different molecular orbitals from the ground state configuration. In the excited electronic state, dynamical changes to the molecular structure (i.e., the atomic framework) are driven by changes in the electronic orbital occupancies, but nuclear motion can also in turn induce changes in electronic states for the molecule (breaking down the Born–Oppenheimer approximation). Together, these nuclear and electronic dynamics might cause bond breaking (photodissociation), isomerization, intersystem crossing (with a change in electron spin), or conversion of electronic energy into excess vibrational energy. Chemical reactions of the resulting new species can then ensue, in competition with relaxation and thermalization, for example, by collisions with N_2_ or O_2_ in air. While the atmosphere is typically thought of as a gaseous environment, there is growing recognition of the role of small particles dispersed in air (i.e., atmospheric aerosols), which provide solid or liquid surfaces and bulk environments where modified chemistry can occur.^35−40^ The theoretical and experimental advances discussed during the CECAM workshop address all these aspects of atmospheric photochemistry, as described below.

## 2 Laboratory Studies of Atmospheric Photochemistry

There are many well-established laboratory protocols to measure wavelength-dependent absorption cross-sections for gaseous molecules and free radicals, photochemical quantum yields, and pressure- and temperature-dependent bimolecular rate coefficients. These methods are described extensively in the scientific literature and will not be reviewed in detail here, but we note some of the experimental challenges that must be overcome.

### 2.1 Absorption Spectra

Measurement of absorption cross-sections typically uses steady-state spectroscopic analysis based on the Beer–Lambert law, which obtains the wavelength-dependent absorbance (*A*(λ)) from changes in the intensity of different wavelengths of light passing through a sample. The absorbance is readily converted to an absorption coefficient (α(λ)) from knowledge of the optical path length (*L*) through the sample: *A* = α*L*. Cavity ring-down spectroscopy (CRDS) also determines absorption coefficients, but from analysis of the rate at which light intensity decays from a high-finesse optical cavity containing the sample.^41,42^ The final step of conversion of the absorption coefficient into an absorption cross-section requires knowledge of the concentration of the absorbing sample, commonly expressed for gaseous species as a number density (*n*) in molecule cm^–3^: α(λ) = *n*σ(λ). Absorption spectra showing resolved rotational fine structure should be measured at high spectral resolution to obtain accurate σ(λ) values.^8,43^ While the final step in this analysis is often straightforward for stable, volatile molecules simply from a measure of the sample pressure, it is more difficult for involatile compounds or for reactive intermediates typically generated *in situ* with uncertain concentrations by flash photolysis. Examples include recent efforts to measure the wavelength-dependent absorption cross-sections of Criegee intermediates; even for formaldehyde oxide (CH_2_OO), the simplest Criegee intermediate, independent experimental studies obtained different spectral band profiles and cross-section values.^44−48^ Nevertheless, the concentrations of reactive intermediates can be established by other means, and absorption cross-sections can be obtained, albeit with greater experimental difficulty.

For atmospheric chemistry applications, the temperature and pressure dependences of absorption spectra also need to be quantified to account for the effects of altitude on the absorption of solar radiation. Making such measurements in the laboratory to parametrize the *T*, *p*, and λ dependence of absorption cross-sections becomes an arduous process. The contribution to light absorption from molecular complexes, in particular those with water molecules, also needs careful consideration because of the abundance of water vapor in the troposphere and the propensity of water molecules to associate with other species.^49,50^ Much as was discussed above for free radicals, quantifying the number densities of these complexes can be difficult in laboratory spectroscopy experiments.

### 2.2 Photochemical Quantum Yields

Following the absorption of solar actinic wavelengths by trace atmospheric constituents such as VOCs, various photochemical outcomes are possible, including bond dissociation to produce fragment free radicals or molecules, in competition with radiative decay by fluorescence, collisional quenching, and other relaxation pathways. Branching between these competing outcomes can be usefully quantified by quantum yield values. Laboratory measurement of product quantum yields requires quantitative detection of specific photoproducts, ideally as a function of actinic wavelength, temperature, and pressure to account for altitude dependent photochemistry in the atmosphere. For example, dissociation quantum yields will be pressure dependent if bond-breaking must compete with collision quenching of excess internal energy, as is observed in some carbonyl compounds.^51^ Such determinations are possible, for example, with FTIR spectroscopic measurement of stable photoproducts, but more-involved methods such as laser-induced fluorescence (LIF), laser ionization, or CRDS are needed to quantify quantum yields for radicals or excited-state atomic and molecular products.^52,53^ The accurate measurement of O(^1^D) quantum yields from ozone photodissociation at wavelengths longer than 290 nm is an important example because of the tropospheric significance of the O(^1^D) + H_2_O → OH + OH reaction as a source of OH radicals.^54^ Even quantum yields of only a few percent for minor photochemical channels can be significant in an atmospheric context, for example, if the precursor compounds are growing in use (and hence emissions to the atmosphere) and the products are long-lived greenhouse gases. A topical case study is the posited production of HFC-23 (fluoroform, CF_3_H) from UV photolysis of CF_3_CHO which is an intermediate in the OH-initiated oxidation of hydrofluoroolefins (HFOs) being introduced as new refrigerant gases.^55,56^ Although existing databases have compiled quantum yield data of atmospheric photochemical significance from available laboratory measurements,^10,11^ these data sets are sparse.^27^

### 2.3 Reaction Rate Coefficients

Flow tubes or reaction chambers isolated from ambient air are commonly used to study the rates of bimolecular gas-phase chemical reactions and their dependence on the pressure and temperature. For example, the kinetics of reactions of OH radicals,^57^ Cl atoms,^58^ NO_3_ radicals,^59^ ozone,^60,61^ and various stabilized Criegee intermediates^62−68^ with VOCs and other co-reactants have been extensively investigated using a range of methods to detect the time scales for loss of one or other reactant. Under experimental conditions in which a volatile molecular reactant is in excess over a radical species, pseudo-first-order kinetic analysis can be used to determine bimolecular rate coefficients without needing to know the concentration of the radical. More challenging is the determination of rate coefficients for radical + radical reactions,^69^ radical + Criegee intermediate reactions,^63,70^ or reactions involving molecular complexes such as those with water molecules (such as so-called “chaperone” mechanisms).^71,72^ Nevertheless, examples exist in the literature of rate coefficient measurements for all of these reaction types. However, a complete picture of the chemistry should include identification of products and their branching ratios, valuable information that is needed for atmospheric chemistry models. Product identification benefits greatly from modern techniques such as multiplexed photoionization and mass spectrometry (MPIMS),^65,73−76^ frequency combs,^77,78^ or chirped pulse Fourier transform microwave spectroscopy.^79^ Atmospheric simulation chambers equipped with spectroscopic and other analytical instrumentation for identification of reaction intermediates and products provide a bridge between controlled laboratory studies of the kinetics of individual reactions and the complex oxidation chemistry occurring in the troposphere.^80^

### 2.4 Aerosols

Recent years have seen substantial advances in experimental laboratory capabilities to study the optical properties of aerosol particles of atmospheric significance.^81−92^ Accurate measurements of the real and imaginary components of particle refractive indices are needed to quantify the contributions to radiative forcing and, hence, climate change from the atmospheric direct effect of different aerosol types. Examples of experimental methods include Raman spectroscopy;^93,94^ single-particle CRDS measurements of extinction, scattering and absorption cross-sections;^81−86,90,95^ photoacoustic spectroscopy of single particles and mobility-selected ensembles;^87,88,90^ and broadband white-light scattering spectroscopy.^89,91,92^ Single-particle mass spectrometry can also characterize the chemical composition of aerosols, either in the laboratory or sampled directly from the atmosphere in field measurements.^96,97^ However, atmospheric aerosol particles are diverse in their sizes, shapes, composition, and morphology, which makes comprehensive study a daunting prospect. Intriguing questions about photochemistry in aerosol particles are attracting growing attention; for example, what are the consequences of high surface-area-to-volume ratios, surface composition and solute enrichment, electric fields at the surfaces of water droplets, supersaturated concentrations of solutes, photocatalytic molecular aggregates, changes in pH, and the nanofocusing of light to give photochemical hotspots in droplets?^17,18,88,98−101^ Might there be polaritonic effects in sub-micrometer diameter aerosol particles, which can act as high quality resonators for specific wavelengths of light? The single-particle spectroscopy methods mentioned above and new tools such as X-ray microscopy^98^ and X-ray absorption and photoelectron spectroscopies (XAS and XPS)^99,102,103^ are beginning to provide answers.

The oxidation of VOCs in the atmosphere produces oxygenated organic compounds, which, because they are more polar and of lower volatility, can condense from the gas phase and contribute to the growth of secondary organic aerosol (SOA) particles. Growing evidence points to reactions of VOCs with Criegee intermediates, and autoxidation reactions initiated by addition of OH to alkenes, providing rapid routes to highly oxygenated molecules (HOMs) which can accumulate in SOA particles.^63,65,104−108^ Laboratory studies of the rates and products of such reactions and of new-particle SOA nucleation and growth, the latter in simulation chambers equipped with particle counters, are now unravelling this complicated chemistry. Sunlight-induced oligomerization of tropospherically photoactive compounds such as pyruvic acid and other α-keto acids dissolved in or at the surface of aqueous aerosol droplets is another proposed route to SOA formation supported by recent laboratory investigations.^109−112^ As a further illustration of the importance of interfacial chemistry in aerosols, pyruvic acid was also recently shown to undergo condensation reactions at the air–water interface of aqueous droplets, forming zymonic acid under dark conditions.^113^

Although not yet implemented directly in experiments on micrometer-scale water droplets, ultrafast transient absorption (TA) spectroscopy studies of photochemical reactions in bulk solutions can provide insights about aqueous photochemistry in atmospheric organic aerosols. Recent examples include measurements using both broadband UV–visible and time-resolved infrared (TRIR) spectroscopy of nitroaromatic compounds (nitrobenzene and nitrophenols),^114^ which are important chromophores in brown carbon aerosols produced by biomass burning.^115^ These studies benefit from time resolution that extends from sub-picosecond to microsecond, allowing multiple, sequential steps in a photochemical reaction to be observed in a single set of experimental measurements, thereby giving a comprehensive picture of the mechanisms of formation of photoproducts and competitive recovery of parent molecules. Analysis of ground-state bleaching features in TRIR spectra can then reveal quantum yields for photochemical loss of the nitroaromatic compounds.

Inorganic aerosols present in the Earth’s atmosphere originate from a variety of sources including volcanic emissions, wind-blown dust, cosmic dust, and oxidation of sulfur-containing compounds to H_2_SO_4_. Recent mass spectrometric analysis of stratospheric aerosols has shown that, in addition to ablation from meteors, sulfuric acid particles at these altitudes contain metals vaporized from rockets and satellites re-entering the atmosphere.^114^ Photochemical and kinetic studies of reactions at the surfaces of such inorganic particles, as well as the kinetics of reactions of gaseous metal atoms and metal-containing molecules, are therefore also targets for laboratory and modeling studies to improve our understanding of the chemistry of the stratosphere and mesosphere and the impacts of human activity on these sensitive regions.^4,116^

## 3 Theoretical and Computational Atmospheric Photochemistry

Section 2 offered an overview of laboratory-based experimental approaches to determine parameters that quantify the rates and efficiencies of photochemical pathways in molecules, reactive intermediates, and aerosol particles of importance in atmospheric chemistry. As was noted earlier, the complexity of VOC oxidation chemistry in the troposphere exceeds both the capabilities and the capacity of existing laboratories for comprehensive study. In this section, we therefore examine how recent advances in computational photochemistry might help to address this capacity challenge and provide data for systems not amenable to experimental measurement. To set the scene, we first review some key concepts in theoretical descriptions of electronic states and the interactions between them that are central to current understanding of nonadiabatic photochemical pathways.

### 3.1 Theoretical Photochemistry

The time-dependent molecular Schrödinger equation,
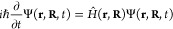
2dictates the time evolution of the molecular wave function Ψ(**r**,**R**,*t*) under the influence of the molecular Hamiltonian *Ĥ*(**r**,**R**) (in a nonrelativistic framework). Here, **r** represents a collective variable for the 3*N*_e_ coordinates of the *N*_e_ electrons composing the molecule, while **R** is a collective variable for the 3*N*_n_ nuclear coordinates. The molecular Hamiltonian *Ĥ*(**r**,**R**) is given by
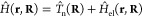
3with being the kinetic energy operator for each nucleus ν with corresponding mass *M*_ν_ and ∇_**R**_ν__^2^ being the second-order differential operator with respect to the nuclear position **R**_ν_. *Ĥ*_el_(**r**,**R**) is the electronic Hamiltonian and is composed of the kinetic energy operator for the electrons and all the Coulombic potential operators (electron–electron, electron–nucleus, nucleus–nucleus). The molecular wave function is often expressed in a given *representation* that simplifies its treatment. One of the most used representations consists of expanding the molecular wave function in a basis of known electronic states. This strategy, suggested by Born and Huang in 1954 and coined the *Born–Huang representation*,^117^ uses the eigenfunctions of the electronic Hamiltonian as a typical basis:
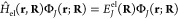
4

Equation 4 represents the (time-independent) electronic Schrödinger equation, and finding the best approximations to its eigenvalues and eigenfunctions is the central goal of quantum chemistry. It is critical to realize that eq 4 is only evaluated for a specific nuclear configuration **R**. As a result, eq 4 returns electronic energies for all electronic states *J* at this particular nuclear configuration **R**, *E*_*J*_^el^(**R**), and the corresponding electronic wave functions Φ_*J*_(**r**;**R**). In other words, the nuclear position acts as a parameter in eq 4, as symbolized by the use of a semicolon “;”. Solving eq 4 for all possible nuclear configurations of a given molecule would allow us to recover the full **R**-dependence for the electronic eigenfunctions and energies (for any electronic states). The electronic energies as a function of **R** are commonly called potential energy surfaces (PESs). The electronic (orthonormal) set of eigenfunctions obtained from this strategy can be used to expand the molecular wave function within the Born–Huang representation:
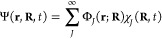
5

The Born–Huang representation of the molecular wave function is formally exact. This representation describes the molecular wave function as a product of a (time-independent) electronic wave function, Φ_*J*_(**r**;**R**), with a time-dependent nuclear wave function, χ_*J*_(**R**,*t*), summed over all electronic states. Inserting eq 5 into the time-dependent Schrödinger equation (eq 2), left multiplying the result by Φ_I_^*^(**r**;**R**), and integrating over the electronic coordinates leads to a set of coupled equations of motion for the nuclear wave functions:
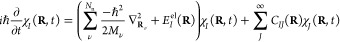
6

The time-evolution of each nuclear wave function (for each electronic state *I*) is dictated by an equation of motion like eq 6. Solving the coupled set of equations of motion given by eq 6 (for each nuclear wave function) is strictly equivalent to solving the time-dependent Schrödinger equation for the molecular wave function (eq 2). Dissecting eq 6 leads to the picture of photochemistry depicted in Figure 1: the first two terms in the right-hand side of eq 6 describe the adiabatic evolution of the nuclear wave function χ_*I*_(**R**,*t*) in the electronic state *I*; the nuclear wave function evolves under the influence of the nuclear kinetic energy operator and the PES for the electronic state *I*, *E*_*I*_^el^(**R**). If only these two terms were to be considered, i.e., *C*_*IJ*_(**R**) = 0 for any *I* and *J*, the nuclear wave function would evolve *solely* in the electronic state *I* and would not be able to change electronic state due the nuclear motion: this defines the well-known (adiabatic) *Born–Oppenheimer approximation*.^118^ The *C*_*IJ*_(**R**) terms act as a source and sink of nuclear amplitude for the nuclear wave function in electronic state *I* and lead to *nonadiabatic* processes resulting from the coupling between nuclear and electronic motion:
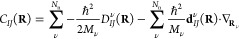
7

The **d**_*IJ*_(**R**) terms are (first-order) nonadiabatic coupling vectors, **d**_*IJ*_(**R**) = ⟨Φ_*I*_(**r**;**R**)|∇_**R**_|Φ_*J*_(**r**;**R**)⟩_**r**_, with ∇_**R**_ the nuclear derivative operator and ⟨···⟩_**r**_ an integration over the electronic coordinates. These nonadiabatic coupling vectors translate the extent to which nuclear motion can couple to different electronic states. The second-order nonadiabatic coupling terms, *D*_*IJ*_(**R**), are given by *D*_*IJ*_(**R**) = ⟨Φ_*I*_(**r**;**R**)|∇_**R**_^2^|Φ_*J*_(**r**;**R**)⟩_**r**_. Hence, moving beyond the Born–Oppenheimer approximation and including the *C*_*IJ*_(**R**) terms in the nuclear dynamics allows the description of internal conversion processes, where a nuclear wave function evolves on a given (time-independent) PES and can transfer its nuclear amplitude to a different electronic state due to the action of the nonadiabatic coupling terms. To describe intersystem crossing processes, an (approximate) spin–orbit coupling Hamiltonian can be included in the molecular Hamiltonian and will also lead to a transfer of the nuclear amplitude between electronic states. Hence, the schematic depiction of a photochemical process given in Figure 1 is a direct product of using the Born–Huang representation to represent the time-dependent molecular wave function.^119^

#### 3.1.1 Adiabatic and Diabatic Representations

A key concept in theoretical photochemistry that may create confusion is the *representation* of the electronic states, namely, *adiabatic* and *diabatic* electronic states. These representations do not change the resulting observables calculated for the molecular system (the Born–Huang representation can formally be expressed either in a basis of adiabatic or diabatic electronic states), but they lead to different interpretations of the same process, here nonadiabatic processes. We offer in the following a brief clarification on this terminology and refer the interested reader to refs (120−124) for more formal (and mathematically precise) discussions.

*Adiabatic* electronic states denote the electronic states obtained by solving the electronic Schrödinger equation as defined in eq 4. In other words, the adiabatic electronic energies and adiabatic electronic wave functions are the eigenvalues and eigenfunctions of the electronic Hamiltonian. The adiabatic electronic states do not cross in energy but can become degenerate, forming the well-known conical intersections that act as funnels between two adiabatic electronic states. As adiabatic electronic states do not cross, their labeling is only dictated by their energy order when solving the time-independent electronic Schrödinger equation: S_*J*_ with *J* = 0, 1, 2, ..., *n* for *E*_0_^el^(**R**) ≤ *E*_1_^el^(**R**) ≤ *E*_2_^el^(**R**) ≤ ...*E*_*n*_^el^(**R**). Hence, the label “S_0_” means the adiabatic electronic state of lowest energy for any nuclear configuration, and the label “S_1_” means the first excited adiabatic electronic state for any nuclear configuration.

*Diabatic* electronic states are connected to the *electronic character* of a given electronic state (e.g., nπ*, ππ*, etc.) and, as such, are not eigenfunctions or eigenvalues of the electronic Hamiltonian. Diabatic electronic states can cross in energy. Importantly, diabatic states can only be defined exactly for diatomic molecular systems, and only quasi-diabatic states can be produced for larger molecules.^125^

A prototypical example where the notions of adiabatic and diabatic representations come into play is the photochemistry of alkali halides such as sodium iodide, NaI. The two lowest diabatic states of this molecule exhibit ionic (purely bound) and covalent (purely dissociative) electronic character. In the adiabatic ground electronic state, the molecule exhibits ionic character close to its equilibrium bond length, but upon increasing the interatomic distance, sodium iodide switches its electronic character to covalent, leading to the well-known dissociation limit for these molecular systems. The first adiabatic excited state of NaI exhibits covalent character near the equilibrium interatomic distance and preserves its bound character at large distances due to a switch to ionic character. The photochemistry of alkali halides (and the interplay between electronic characters) was thoroughly studied with ultrafast spectroscopy and associated theory.^126−128^

**Figure 2:**
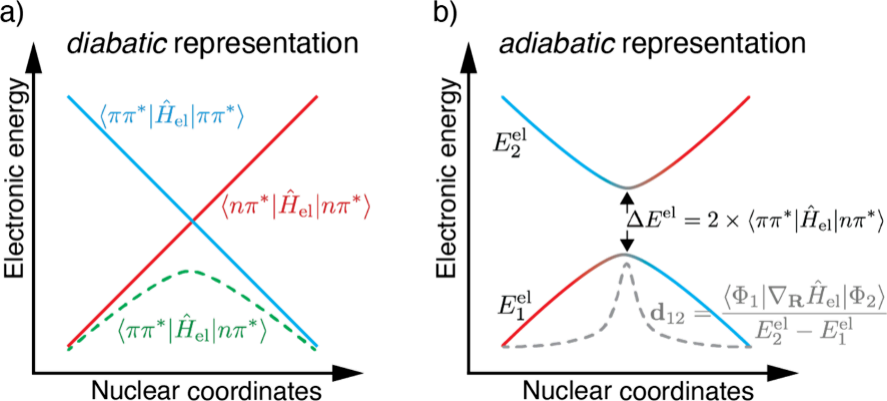
Schematic depictions of (a) diabatic and (b) adiabatic representations for the electronic states.

The following text provides a brief discussion of the (mathematical and conceptual) connections between adiabatic and diabatic electronic states. Taking an example illustrative of carbonyl photochemistry in the troposphere, we consider here a VOC bearing an enone moiety with two diabatic states exhibiting nπ* and ππ* electronic character, denoted by |nπ*⟩ and |ππ*⟩. The electronic energies of these two diabatic states are given by the expectation values for the electronic Hamiltonian, i.e., ⟨nπ*|*Ĥ*_el_|nπ*⟩ and ⟨ππ*|*Ĥ*_el_|ππ*⟩, and are depicted schematically in Figure 2a (red and blue lines, respectively) in terms of some nuclear coordinates describing the shape of the molecule. The two diabatic electronic energy curves can cross. The *diabatic coupling* between these two diabatic states is given by ⟨nπ*|*Ĥ*_el_|ππ*⟩ = ⟨ππ*|*Ĥ*_el_|nπ*⟩ (dashed green curve in Figure 2a). The diabatic coupling is large when the two diabatic electronic states are strongly coupled via the electronic Hamiltonian, i.e., when the two diabatic states strongly interact. The different mathematical terms discussed above are the elements of the electronic Hamiltonian operator expressed in a diabatic basis:
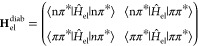


We note that the nuclear kinetic energy operator matrix, **T**_n_^diab^, is diagonal in the diabatic basis (and required to form the full molecular Hamiltonian **H**^diab^ = **T**_n_^diab^ + **H**_el_^diab^). As stated above, the adiabatic electronic states are the eigenstates of the electronic Hamiltonian and therefore make the electronic Hamiltonian diagonal. Denoting the two adiabatic states as |Φ_1_⟩ and |Φ_2_⟩, we then have that ⟨Φ_1_|*Ĥ*_el_|Φ_1_⟩ = *E*_1_^el^, ⟨Φ_2_|*Ĥ*_el_|Φ_2_⟩ = *E*_2_^el^, and ⟨Φ_1_|*Ĥ*_el_|Φ_2_⟩ = ⟨Φ_2_|*Ĥ*_el_|Φ_1_⟩ = 0. *E*_1_^el^ and *E*_2_^el^ are the adiabatic electronic energies (depicted in Figure 2b). The electronic Hamiltonian expressed in an adiabatic basis is therefore diagonal,
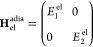
while the nuclear kinetic operator matrix in this basis, **T**_n_^adiab^, is not. The off-diagonal elements of the nuclear kinetic energy operator in the adiabatic basis are related to the first- and second-order nonadiabatic coupling terms discussed above (Section 3.1). We finally note a connection between the diabatic and adiabatic worlds by expressing the adiabatic electronic energies (eigenvalues of the electronic Hamiltonian) in terms of the diabatic matrix elements:
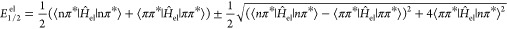
8This expression reveals the interconnections between the diabatic matrix elements and the adiabatic electronic states and will be used further below.

The scheme provided in Figure 2 helps to summarize the differences between the adiabatic and the diabatic representations. As stated above, the diabatic states carry a given electronic character and their respective energy curves (⟨nπ*|*Ĥ*_el_|nπ*⟩ and ⟨ππ*|*Ĥ*_el_|ππ*⟩) can cross in the nuclear configuration space (Figure 2a). The coupling between these diabatic electronic states is mediated by the diabatic coupling, i.e., off-diagonal element ⟨ππ*|*Ĥ*_el_|nπ*⟩. Conversely, the adiabatic electronic states cannot cross (Figure 2b) and are labeled only by their ordering in the solution of the Schrödinger equation. While the first adiabatic state in Figure 2b has nπ* character for small nuclear coordinates, the same adiabatic electronic state will have ππ* character at larger nuclear coordinates (see red/blue color code in Figure 2b). Hence, it is improper to assign an electronic character to an adiabatic electronic state without specifying the precise nuclear coordinates for which this is true.

Another insight provided by eq 7 is that adiabatic electronic states can be degenerate if and only if ⟨nπ*|*Ĥ*_el_|nπ*⟩ = ⟨ππ*|*Ĥ*_el_|ππ*⟩ and ⟨ππ*|*Ĥ*_el_|nπ*⟩ = 0. These two conditions cannot be fulfilled simultaneously by a single nuclear coordinate, meaning that diatomic molecules can only exhibit avoided crossings between adiabatic potential energy curves.^129^ For polyatomic molecules, these points of degeneracy between adiabatic states can be lifted linearly only along two specific nuclear coordinates (which define the branching space), leading to the appearance of conical intersections. Any other nuclear displacements (referred to as the seam space) would preserve the degeneracy between the adiabatic electronic states. We stress here that conical intersections are a product of the adiabatic representation and do not appear in the diabatic representation.

How can the adiabatic and diabatic pictures offer the same overall description of a molecular wave function while being apparently so different? Let us consider a case where the diabatic coupling between two diabatic states (⟨ππ*|*Ĥ*_el_|nπ*⟩, green dashed line in Figure 2a) is weak. A weak diabatic coupling means that a VOC with an enone moiety, initially photoexcited to its bright |ππ*⟩ diabatic state (at small nuclear coordinates in Figure 2a), will relax toward larger nuclear coordinates by remaining (mostly) in this diabatic state (the photoexcited VOC will follow the blue diabatic curve) as mixing with the other diabatic state (of different electronic character) will be minimal. Equation 7 tells us that, at the nuclear configuration where two diabatic states cross (⟨nπ*|*Ĥ*_el_|nπ*⟩ = ⟨ππ*|*Ĥ*_el_|ππ*⟩), the energy gap between the two resulting adiabatic electronic states, Δ*E*^el^ = *E*_2_^el^ – *E*_1_^el^, is equal to twice the diabatic coupling, 2⟨ππ*|*Ĥ*_el_|nπ*⟩. Hence, a weak diabatic coupling in the diabatic representation when diabatic states cross means a small gap between the adiabatic electronic states (at the diabatic crossing point). Considering that the coupling between adiabatic states is mediated by the nonadiabatic coupling terms and that such terms are inversely proportional to the energy gap between the two (adiabatic) electronic states that they couple, the nonadiabatic coupling terms will be large in the adiabatic representation at the location where the diabatic states would cross. Hence, in the adiabatic representation, the VOC will be photoexcited into the second adiabatic state (having ππ* character at small nuclear coordinates, depicted by the blue portion of *E*_2_^el^ in Figure 2b) and evolves toward the point where *E*_2_^el^ and *E*_1_^el^ come very close in energy (as Δ*E*^el^ = 2⟨ππ*|*Ĥ*_el_|nπ*⟩ is small at the diabatic crossing point), leading to a large nonadiabatic coupling term that will transfer the molecule to *E*_1_^el^, ensuring that the VOC preserves ππ* character at larger nuclear coordinates (also shown as blue in Figure 2b).

Repeating the same scenario, considering this time a VOC with electronic states of the same character as above but with a strong diabatic coupling (due, for example, to the nature of the chromophoric groups), we would have a VOC suffering a change of electronic character during its relaxation. That is, the photoexcited VOC evolves first on the diabatic curve ⟨ππ*|*Ĥ*_el_|ππ*⟩ and then changes to ⟨nπ*|*Ĥ*_el_|nπ*⟩ under the influence of a large diabatic coupling between ππ* and nπ*. In the adiabatic representation, the VOC would remain (adiabatically) in the second adiabatic electronic state due to a large energy gap between the adiabatic state and a small nonadiabatic coupling term. In both cases, the molecule changes its electronic character from ππ* to nπ*. Hence, the diabatic and adiabatic pictures provide the very same outcome for the molecular system.

We finally note that the two-state picture developed above can easily be extended to intersystem crossing processes and spin–orbit coupling, which contribute significantly to the photochemistry of carbonyl compounds in the atmosphere. Equation 4 does not include any relativistic contributions and, as such, does not account for spin–orbit coupling. Hence, the (adiabatic) electronic states obtained from eq 4 for different spin multiplicities behave as spin-diabatic states and their respective electronic energies can cross: the adiabatic electronic states can be assigned a well-defined “spin character”, for example, singlet or triplet. The matrix element of a spin–orbit coupling Hamiltonian with respect to the interaction between a singlet and a triplet adiabatic electronic state acts as a spin diabatic coupling between these two electronic states. Hence, the spin–orbit coupling strength calculated in most quantum-chemical codes should be seen as a (spin) diabatic quantity. It is only upon diagonalization of the spin-diabatic Hamiltonian matrix (including spin–orbit coupling) that spin-adiabatic electronic states can be obtained, for which the (electronic) spin quantum number is no longer a “good” quantum number because these resulting spin-adiabatic electronic states are a mix of spin multiplicities through the action of the spin–orbit coupling. In other words, the (spin) character of the electronic states is mixed in the spin-adiabatic representation.^130^

### 3.2 Applications of Theoretical Photochemistry to Molecules

Simulating the photochemistry of a given molecule requires the use of nonadiabatic molecular dynamics methods, which offer a (often approximate) numerical solution to the time-dependent Schrödinger equation expressed within the Born–Huang representation (eq 6). While nonadiabatic molecular dynamics simulations give access to the mechanistic details of internal conversions (and can depict intersystem crossings too), they require electronic-structure information such as electronic energies, nonadiabatic (or diabatic) coupling terms, nuclear gradients, and sometimes Hessians for different regions of the configuration space visited by the photoexcited molecule. Hence, nonadiabatic molecular dynamics simulations always require electronic-structure (or quantum-chemical) calculations for the propagation in time of the nuclei. In the following, we briefly mention the central methods discussed during the CECAM workshop in the context of atmospheric chemistry.

A computational study of a photochemical reaction almost always begins by mapping electronic energies over the nuclear distortions describing the photodynamics of the molecules. Calculating electronic energies as a function of nuclear coordinates, commonly known as potential energy surfaces, also allows conical intersections to be located and their branching and seam space to be determined. A similar approach can be used for singlet–triplet crossings. Such mappings of the PESs are key to (i) benchmarking the level of electronic-structure theory and (ii) picking the best compromise between efficiency and accuracy, as nonadiabatic molecular dynamics simulations require a large number of electronic-structure calculations.

In atmospheric photochemistry, PESs are often mapped with multireference methods like MRCI (multireference configuration interaction) or (X)MS-CASPT2 (multistate complete active space second order perturbation theory) when photodissociation occurs.^131−134^ Photodissociations are indeed known to challenge simpler (single-reference) excited-state quantum-chemical methods like LR-TDDFT (linear-response time-dependent density functional theory),^135−137^ EOM-CCSD (equation-of-motion coupled cluster singles and doubles),^138^ or ADC(2) (algebraic diagrammatic construction to second order).^139^ The OH photodissociation channel of *tert*-butyl hydroperoxide discussed during the CECAM workshop offers an example of the challenge caused by such processes for methods like LR-TDDFT and ADC(2).^140^ However, these methods can offer an interesting alternative for the simulation of photochemical reactions involving activated processes, i.e., systems with long-time dynamics in the excited electronic states, but only after careful benchmarking of their behavior beyond the Franck–Condon region. It is worth pointing out that ADC(2) (and the underlying MP2 calculations for the ground state) predicts artificially low-energy crossing regions between the first excited state (with *n*π* character) and the ground state of carbonyl-containing molecules,^141^ which hampered proper descriptions of the nonradiative decays of VOCs like pyruvic acid or 2-hydroperoxypropanal. Other alternative approaches, like hh-TDA (hole–hole Tamm–Dancoff approximation)^142^ or FOMO-CASCI (floating occupation molecular orbital complete active space configuration interaction),^143^ were discussed during the workshop for studying the photodynamics of *o*-nitrophenol and offer an attractive compromise between efficiency and a proper description of conical intersections between the ground and first excited electronic states.

Once the PESs have been mapped and an adequate level of electronic-structure theory is found, a more detailed study of the photodynamics of VOCs can begin. Any nonadiabatic molecular dynamics methods will require the definition of a set of initial conditions (nuclear positions and nuclear velocities) that will be used to mimic the photoexcitation process undergone by the molecule and initiate the dynamical simulations.^144,145^ The ground-state (nuclear) probability density of the molecule of interest is usually sampled by using either a harmonic Wigner distribution or different flavors of ab initio molecular dynamics. Different works have focused on the importance of adequately sampling initial conditions for the simulation of atmospheric photochemistry.^146,147^ We stress here one aspect discussed during the workshop: the limitation of the harmonic Wigner distribution for (flexible) molecules having low-energy vibrational modes that might be photoactive, exemplified by the photodynamics of methylhydroperoxide, for which an adequate description of the ground-state probability density could only be recovered by using ab initio molecular dynamics combined with a quantum thermostat.^147^ We will discuss below how this sampling of initial conditions can also be used to predict photoabsorption cross-sections (as further discussed in Section 3.3).

**Figure 3:**
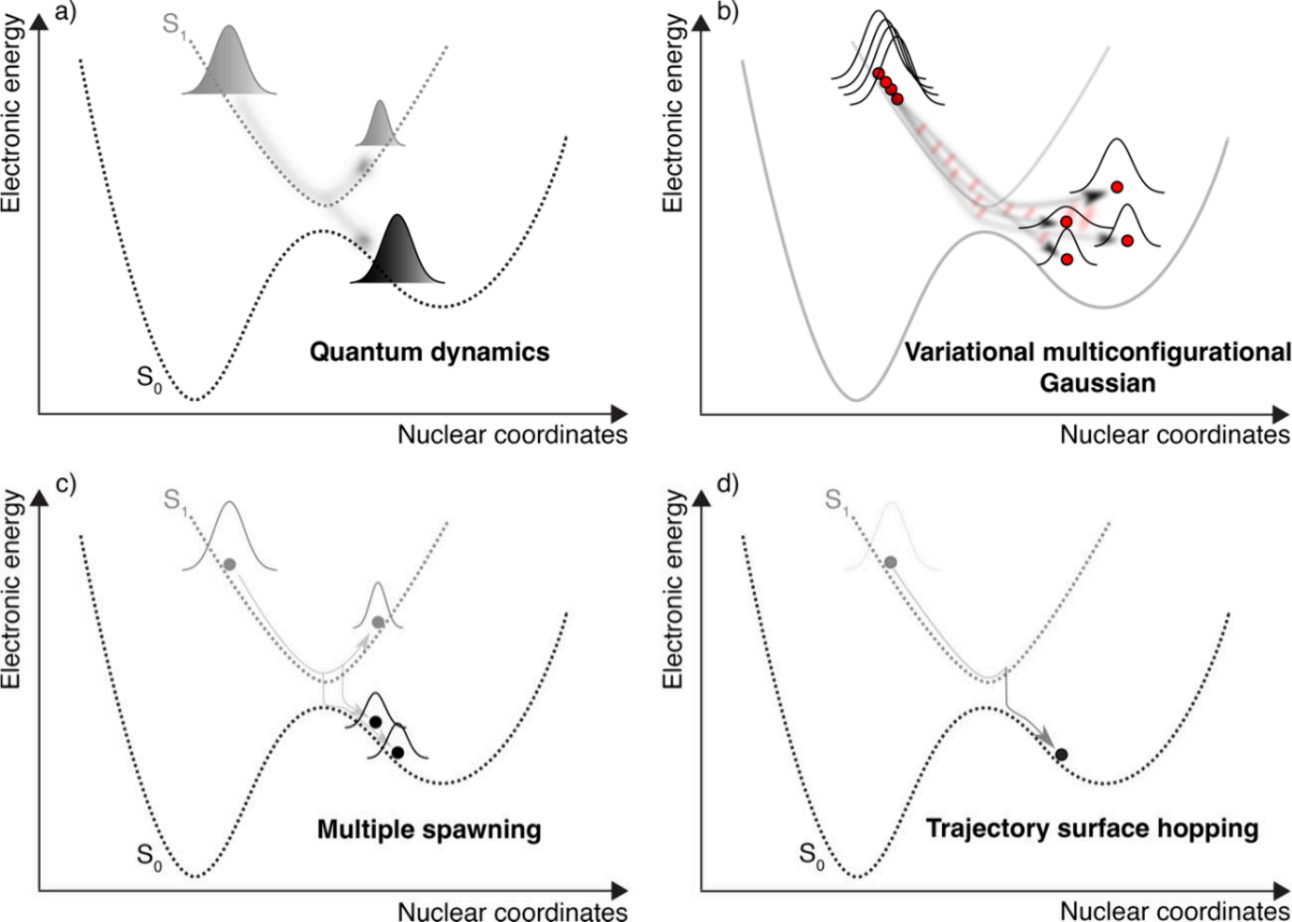
Schematic depictions of different nonadiabatic molecular dynamics strategies. (a) Quantum dynamics, (b) variational multiconfigurational Gaussian, (c) full or ab initio multiple spawning, and (d) trajectory surface hopping.

Nonadiabatic molecular dynamics simulations can then be performed to obtain mechanistic information about the photodynamics of a given atmospheric molecule, in particular, the formation of photoproducts and their respective quantum yields. A nonadiabatic molecular dynamics simulation is sensitive to the level of electronic-structure theory^148^ and sampling of initial conditions,^147^ as stressed during the CECAM workshop, and relies on a prior careful assessment of their quality, as discussed above. Different nonadiabatic molecular dynamics strategies are illustrated in Figure 3 and were discussed during the workshop.^149^ Quantum dynamics (Figure 3a), like MCTDH (multiconfiguration time-dependent Hartree),^150−153^ constitute the gold standard because all nuclear quantum effects are accurately described in the dynamics by solving eq 6 in the diabatic representation on a grid or using (time-dependent) single-particle functions in MCTDH. The computational cost of such techniques often limits the number of nuclear degrees of freedom that can be considered for a given molecule; instead, simulations typically adopt a model Hamiltonian (for example, a vibronic coupling model^154^) to incorporate the most important nuclear degrees of freedom for the nonadiabatic dynamics.

MCTDH has served as a framework for subsequent method development like G-MCTDH (Gaussian MCTDH) and vMCG (variational multiconfigurational Gaussian),^155,156^ which propose to describe the dynamics of the nuclear wave functions in a basis of traveling multidimensional coupled Gaussian functions propagated fully variationally. The advantage of the vMCG strategy (Figure 3b) resides in its compatibility with on-the-fly (or direct-dynamics) simulations, meaning that the electronic-structure quantities are not precalculated prior to propagation (as would be done in MCTDH) but along the dynamics of each of the multidimensional Gaussians. Direct-dynamics vMCG (DD-vMCG) requires Hessians for the propagation of the nuclear wavepackets and resorts to a database to store and interpolate electronic-structure quantities,^157^ alleviating significantly the computational effort associated with this type of nonadiabatic dynamics. AIMS (ab initio multiple spawning) also represents the nuclear wave functions in eq 6 in a basis of traveling, coupled multidimensional Gaussians, but in AIMS the Gaussians are propagated classically and their number can be increased (thanks to the spawning algorithm) to describe the transfer of nuclear amplitude in nonadiabatic regions adequately (Figure 3c).^145,158,159^ Being a practical realization of the formally exact technique FMS (full multiple spawning),^160−162^ AIMS relies on a series of approximations to be compatible with on-the-fly dynamics and cannot describe fine nuclear quantum effects such as tunnelling without an adaptation of its spawning algorithm.

All the methods described so far emerge from a derivation based on first-principles and can, in principle, be converged to a numerically exact solution of the molecular time-dependent Schrödinger equation. Conversely, mixed quantum/classical methods propose to propagate the nuclei using classical dynamics, but these dynamics will be influenced by nonadiabatic effects obtained from a quantum propagation of the electrons in support of the classical nuclear trajectory.^144^ These methods target, by construction, an approximation to eq 6, and the most celebrated member of this family is TSH (trajectory surface hopping).^163−165^ In TSH, the nuclear probability densities are represented by a swarm of independent classical trajectories. Each TSH trajectory evolves adiabatically in a given electronic state and can hop to a different electronic state (mostly in regions of strong nonadiabatic interactions) by application of a stochastic algorithm (Figure 3d). In the most commonly used version of TSH, fewest-switches TSH,^164^ the stochastic algorithm is based on probabilities calculated from the electronic wave function propagated along the (independent) trajectory and sensitive to nonadiabatic events. TSH requires a large number of independent classical trajectories to obtain converged results, in particular for photoproducts with a low quantum yield.^166^ The original version of TSH suffers from issues with the description of decoherence when nuclear wavepackets branch at an intersection,^167^ but efficient corrections have been developed to fix this issue in molecular simulations.^168,169^ Other mixed quantum/classical techniques have been developed over the years to overcome the limitations of TSH,^170^ simplify the treatment of nonadiabatic transitions (including spin–orbit coupling),^171^ and extend the applicability of these methods to processes involving long-time coherence.^172,173^

A final aspect to consider for the formation of photoproducts is the importance of nonstatistical (or athermal) effects in the ground electronic state after the molecule relaxes nonradiatively from its excited electronic states via one or more conical intersections. The nonadiabatic relaxation can lead to the formation of ground-state molecules with internal energies deviating significantly from a Boltzmann distribution at early times, which can result in athermal reactivity and the formation of (sometimes) unexpected products.^174^ Such processes were discussed in the context of gas-phase photochemistry^175,176^ and also observed for nitroaromatic compounds of atmospheric importance.^177^

The electronic-structure methods and nonadiabatic molecular dynamics strategies discussed above are implemented in different software packages. Surface hopping can be performed with Newton-X,^178^ SHARC,^179^ ABIN,^180^ JADE,^181^ or Quantics.^182^ Quantics can also be used for MCTDH and vMCG. FMS90 is a code for AIMS and is part of the Molpro package.^183^ The COSMOS project^184^ was announced during the CECAM workshop and aims to transform Quantics into a general platform for calculation and analysis of energy- and time-resolved observables via any nonadiabatic molecular dynamics method. During the CECAM workshop, other emerging opportunities were discussed to lower the computational burden of nonadiabatic molecular dynamics, particularly for the electronic structure, but also using machine-learning approaches to predict long-time dynamics from short-time simulations^185^ or analogue quantum computers to simulate chemical dynamics and vibronically resolved absorption spectra.^186,187^

### 3.3 Calculation of Absorption Spectra for Atmospheric Molecules

Recent progress in the efficient computational calculation of molecular absorption spectra in the UV and visible regions is illustrated by the remarkable agreement with experimental measurements for a variety of organic compounds present in the atmosphere. This agreement applies to both the wavelengths at which molecules absorb and their wavelength-dependent absorption cross-sections, σ(λ), which are a measure of how strongly the molecules absorb light. This progress derives from improvements to electronic structure theory methods for calculation of ground- and excited-state electronic energies and transition dipole moments and to advances in techniques to improve the quantum nuclear effects of a molecule in the ground electronic state.

**Figure 4:**
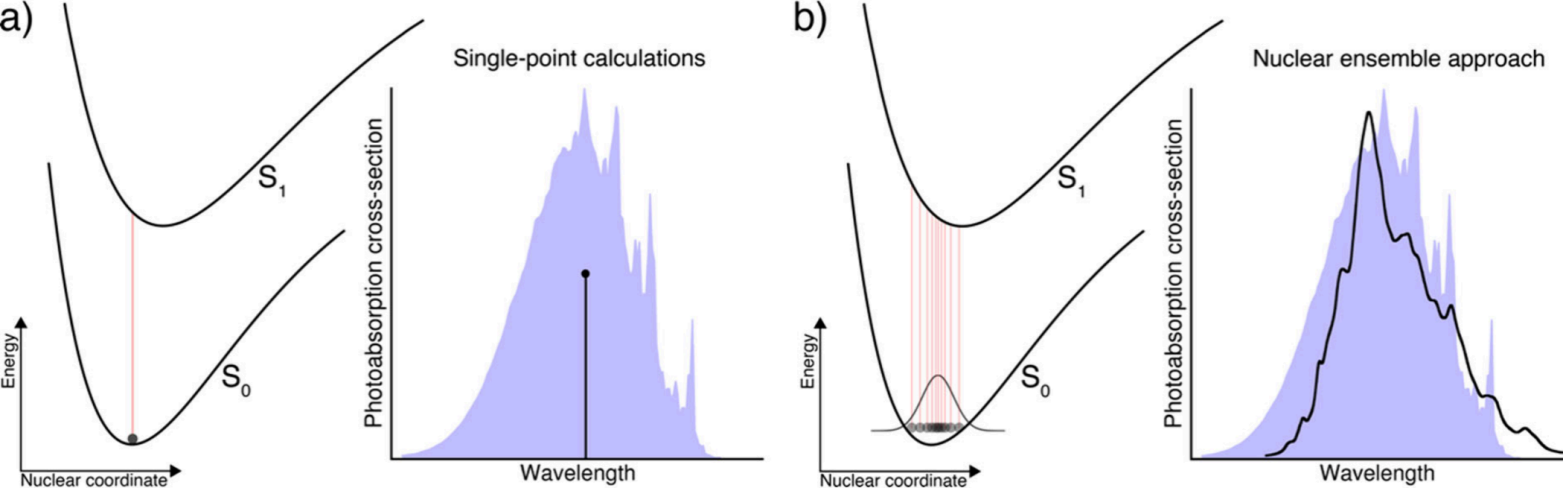
Calculating the photoabsorption cross-section of a molecule. (a) Single-point calculation: The ground-state minimum-energy geometry of the molecule is located, vertical excitation energies and transition dipole moments are calculated for this nuclear geometry for all excited electronic states considered (left panel), resulting (right panel) in a single “stick” to depict the photoabsorption cross-section; the blue shaded area represents the experimental spectrum. (b) Nuclear ensemble approach: A ground-state probability density is approximated for the molecule (chosen from harmonic Wigner sampling, ab initio molecular dynamics with quantum thermostat, path-integral molecular dynamics) and used to sample molecular geometries (typically 500–10,000). For each molecular geometry, an electronic-structure calculation is conducted to obtain the vertical excitation energies and transition dipole moments. The overall photoabsorption cross-section is obtained by averaging over all (broadened) vertical transitions, recovering the shape and width of each absorption band. Reproduced from ref (188). Copyright 2022 American Chemical Society.

The nuclear ensemble approach (NEA) illustrated in Figure 4 offers a simple strategy to sample ground-state structures representative of the vibrational motions of the molecule and hence to compute the wavelength-dependent shapes of absorption bands.^189,190^ In the CECAM workshop, different flavours of the NEA, which is a numerical realization of the reflection principle,^34^ were presented. These approaches differ in the strategies employed to approximate the ground-state nuclear probability density for molecules: harmonic Wigner distribution,^34,166^ path-integral molecular dynamics,^191^ or *ab initio* molecular dynamics coupled to a quantum thermostat.^192^ A set of representative molecular geometries is sampled from one of these distributions, and vertical excitation energies and transition dipole moments are calculated at each of these nuclear configurations for all excited electronic states considered (Figure 4b). The levels of electronic-structure theory discussed in the CECAM workshop included XMS-CASPT2, ADC(2), LR-TDDFT, FOMO-CASCI, and hh-TDA. Importantly, the NEA recovers non-Condon contributions to the computed spectrum thanks to the sampling of nuclear configurations beyond the ground-state equilibrium geometry, offering a clear advantage over the use of single-point calculations combined with a simple broadening of the vertical transitions (Figure 4a). A general test of the NEA capabilities was recently proposed for different atmospheric VOCs.^188^ The NEA can also be used to predict photoelectron spectra, and this strategy was successfully deployed to identify the photoproducts obtained by UVA absorption of gas-phase pyruvate, in particular an unexpected methide anion, CH_3_^–^.^193^ Jahn–Teller effects in allene were also characterized by the NEA, which was used to simulate photoelectron, X-ray absorption, and Auger spectra.^194^

The workflow of the NEA is not complex *per se* but can become tedious for nonexperts in computational photochemistry. Efforts are therefore being made to automate the NEA and build this technique into openly available computational tools such as AtmoSpec for wider use.^195^

Challenges remain in the accurate computation of the tails of absorption spectra, where overlap with the solar flux in the troposphere is typically greatest. Even for purely dissociative excited electronic states, the tail of the absorption cross-section produced by the NEA tends to depart from the exact result in this region.^188^ As the NEA relies on approximations of the reflection principle, it cannot capture vibronic progressions in a photoabsorption cross-section because the method does not account for the overlap between the initial and final vibrational states of the molecule. Nevertheless, the envelope (intensity and width) of each absorption band is expected to be adequately depicted. Accurate calculation of excited-state properties of reactive species such as Criegee intermediates possessing unusual electronic structures also places considerable demands on the computational quantum chemistry methods needed for NEA calculations of absorption spectra.^48,196,197^ The range of applicability and good practice when using the NEA were recently discussed.^198^

One example of the value of calculating wavelength-dependent absorption spectra is for HOSO_2_ which is an intermediate in the oxidation of SO_2_ to SO_3_ and H_2_SO_4_ in the atmosphere. MS-CASPT2 calculations of the absorption spectrum and hence evaluation of altitude-dependent solar photolysis rates using eq 1 showed how HOSO_2_ → OH + SO_2_ photolysis is faster in the stratosphere than in the troposphere.^199^ In both atmospheric regions, long tropospheric photolysis lifetimes of HOSO_2_ mean that it preferentially reacts with O_2_ to make SO_3_ which is an important contributor to acid rain. However, this HOSO_2_ photochemistry is predicted to be more significant in the atmosphere of Venus where O_2_ concentrations are much lower than on Earth.

### 3.4 Photochemical Quantum Yields

**Figure 5:**
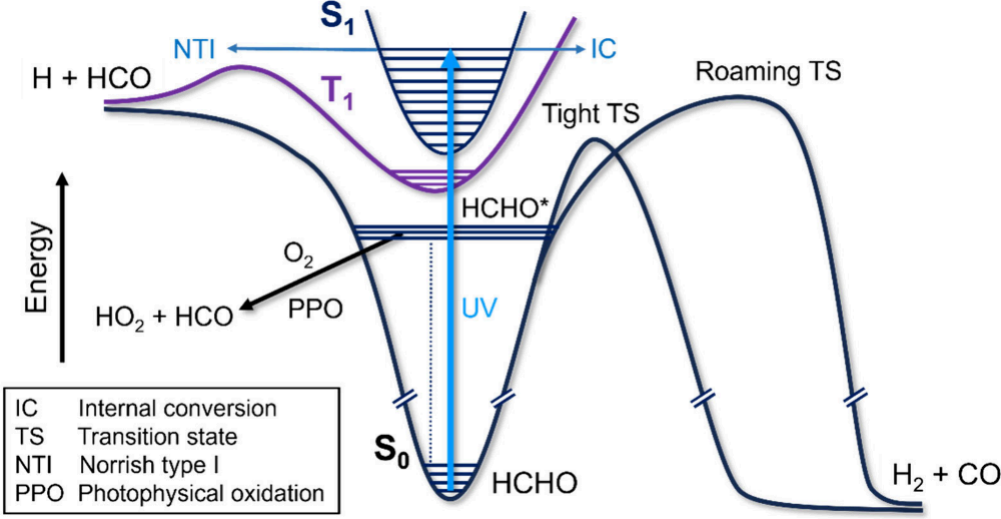
Schematic diagram showing the photochemical pathways for the prototypical carbonyl compound formaldehyde following UV excitation to its S_1_ state. Mechanisms are described in the main text, and abbreviations are defined in the inset key. Horizontal lines indicate some of the quantized vibrational levels in the S_0_, S_1_, and T_1_ electronic states. HCHO* denotes vibrationally excited HCHO in the S_0_ electronic ground state.

The importance of computing accurate photochemical quantum yields and the many challenges still faced by computational chemists are illustrated by atmospheric carbonyl compounds. Figure 5 schematically shows some of these photochemical complexities for the simplest carbonyl compound, formaldehyde.

The initial photoexcitation at long UV wavelengths, commensurate with solar radiation in the troposphere, is via a weak (or forbidden) π* ← n electronic excitation. This transition gains oscillator strength with certain molecular framework distortions, necessitating the use of NEA methods to sample fully the ground state geometries and non-Condon effects in the transition dipole moments. In some cases, including formaldehyde, the π* ← n absorption band is vibrationally (and rotationally for HCHO) structured, meaning that calculations drawing on the reflection principle will not reproduce the correct band contours but may offer a qualitative picture of the band envelope. From the S_1_ state, the carbonyl molecules can rapidly cross to nearby triplet states, often on ultrafast time scales and with high quantum yields. Norrish type I photochemistry in the excited triplet state gives radical photoproducts, whereas relaxation to the ground electronic state and dissociation over a tight transition state produce molecular products. In the case of formaldehyde, one molecular product is H_2_, the yields of which need to be understood because of possible impacts that higher H_2_ concentrations will have on the oxidizing capacity of the atmosphere (hence acting as an indirect greenhouse gas) with the growth of the hydrogen economy.^200^ The branching between the radical and molecular pathways is wavelength dependent, and can be further influenced by large amplitude “roaming” dynamics on the ground state PES that circumvent the tight TS but still form molecular products.^201^ These competing dynamics can occur over extended time scales, which makes simulation and prediction of quantum yields difficult. Moreover, the internally excited ground-state molecules can react with O_2_ to make HO_2_ in so-called “photophysical oxidation” reactions that are energetically inaccessible from thermalized molecules in their vibrational ground-state energy levels.^202^ Yet this photochemistry matters in the atmosphere because carbonyl compounds such as formaldehyde are key intermediates in OH and O_3_ initiated oxidation of VOCs including isoprene, their photochemistry is a source of HO_*x*_ radicals, and in some cases this photochemistry might be a step in unintended pathways to the production of long-lived greenhouse gases such as HFC-23 (CF_3_H) from the oxidation of hydrofluoroolefins.^56,203^

Notwithstanding the complexities of carbonyl photochemistry outlined above, calculation of the nuclear dynamics in photoexcited molecules (as described in Section 3.2) can simulate changes in electronic state and spin and the competition between bond breaking, isomerization, and relaxation to the ground electronic state. In principle, these calculations can therefore predict branching between different photochemical pathways, and hence quantum yields. The photochemical dynamics of several molecules of atmospheric interest have been studied in this way using TSH dynamics; examples of organic compounds include methyl hydroperoxide at ice surfaces,^204^ the chlorofluorocarbon CF_2_Cl_2_,^146^ the simplest Criegee intermediate CH_2_OO,^205^ a fluorinated Criegee intermediate HFCOO,^206^ and the hydrochlorofluorocarbon C_2_H_2_F_3_Cl.^207^

Nonadiabatic dynamics simulations can also be applied to the photochemistry of inorganic compounds containing toxic metal ions. One illustration is the atmospheric cycling of mercury, which can chemically and photochemically switch between Hg(0) and Hg(II) oxidation states and is a major environmental hazard for which remediation will benefit from the insights provided by recent TSH calculations and atmospheric chemistry modeling.^208^ Other metals are being unintentionally introduced into the upper atmosphere through ablation of material from discarded rocket stages and re-entry of rockets or satellites into the atmosphere where they may “burn up”.^114^ The consequences of additional metal loading in this sensitive region of the atmosphere are not well understood, but the sudden appearance of high-altitude sporadic metal layers formed from material ablated from meteors has prompted laboratory and computational studies of atmospheric metal chemistry.^4^

If calculations of *wavelength-dependent* quantum yields are needed, great care is required when selecting the initial conditions for nonadiabatic molecular dynamics. In the context of atmospheric photochemistry, a protocol has been proposed to obtain excitation-energy dependent absorption cross-sections using the NEA (Section 3.3) and split them into different energy windows from which initial conditions can be selected.^209^ Care is required for this first step when the molecule of interest presents photoactive low-energy vibrational modes. Performing nonadiabatic molecular dynamics for each window and determining their resulting population of photoproducts provide access to photoproduct quantum yields for each energy window, a coarse-grained approximation to the wavelength-dependent quantum yields. This strategy was used for different atmospheric applications discussed in the CECAM workshop like *tert*-butyl hydroperoxide (we note that benchmarking TSH with an AIMS run was proposed in this study),^140^ methyl hydroxyperoxide,^147^ pyruvic acid,^210^ CF_3_COCl,^211^ HOSO_2_ and SO_3_,^199^ methyl nitrate,^212^ peroxynitrous acid,^213^ or 2-hydroxypropanal.^214^

**Figure 6:**
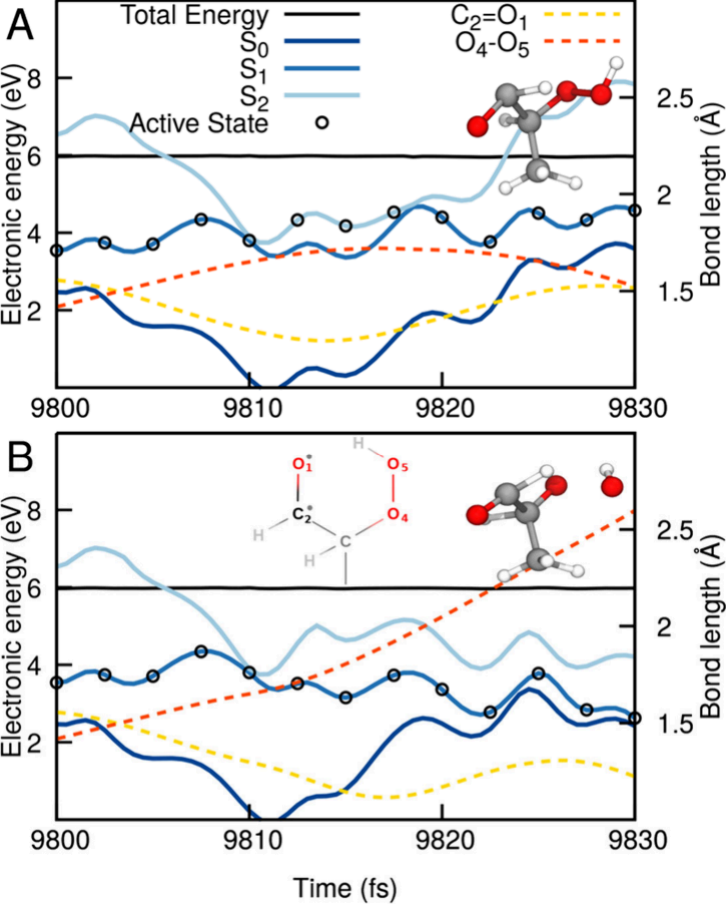
Example trajectories for the excited-state dynamics of 2-hydroxypropanal illustrating the upfunneling, or diabatic trapping, mechanism, which hampers the photodissociation of OH. The energy traces (SCS-ADC(2)/def2-SVP) along the dynamics (between 9800 and 9830 fs following photoexcitation) highlight the three lowest electronic states, S_0_ (dark blue), S_1_ (blue), and S_2_ (light blue), while the driving state for the TSH dynamics is indicated by black empty circles. The total classical energy is given with a black solid line. The length of the O_4_–O_5_ bond of the hydroperoxide moiety is indicated by a red dashed line and the right-hand axis, while the carbonyl C_2_=O_1_ bond length is given by a yellow dashed line. The atom numbering is provided in panel B. Molecular structures illustrating the photoproducts formed are included as insets. Panel A shows a TSH trajectory exhibiting diabatic trapping, at *t* = 9810 fs; the TSH trajectory jumps from S_1_ to S_2_, preserving the n(O)π*(C=O) electronic character of the molecule, before jumping back to S_1_ just before 9820 fs, still preserving the n(O)π*(C=O) character. The O_4_–O_5_ bond (red dashed line) remains intact during this process. Panel B shows the very same trajectory but this time artificially constrained to remain (adiabatically) in the S_1_ electronic state. At *t* = 9810 fs, the S_1_ electronic state adiabatically changes its character from n(O)π*(C=O) to n′(OO)σ*(OO), leading to an immediate photodissociation of the O_4_–O_5_ bond (red dashed line) and a release of OH. Hence, nonadiabatic transitions delay the OH photolysis of 2-hydroxypropanal. Reproduced from ref (214). Copyright 2022 American Chemical Society.

The photochemistry of 2-hydroxypropanal highlights the diversity of photochemical processes that multichromophoric VOCs can undergo, ranging from multiple photoproducts, formed either in the excited states or in the ground electronic state with athermal effects, to upfunneling (or diabatic trapping).^215,216^ This latter process is illustrated in Figure 6 and is typical of flexible multichromophoric molecules where the molecule remains trapped in a given diabatic state due to a very weak diabatic coupling with other states, slowing down the formation of photoproducts.^214^ Diabatic trapping was also observed for another multichromophoric VOC, C_6_-hydroperoxy aldehyde (C6-HPALD).^217^ The nonadiabatic molecular dynamics of C6-HPALD, conducted with TSH, were used to build a nonadiabatic energy-grained master equation model, and both methods provided qualitatively similar dissociation rates for the photorelease of OH.

### 3.5 Reaction Rate Coefficients

One consequence of photochemical dynamics producing reactive intermediates such as free radicals is that these products can undergo further chemical reactions of importance in the atmosphere, the exploration of which increasingly benefits from computational chemistry capabilities.^32^ These reactions can be theoretically described by a ground electronic state PES and application of reaction rate theories such as transition state theory (TST), often with the assumption of thermal reactants for reactions in the lower atmosphere. Whether computed or experimentally measured, reaction rates are incorporated into atmospheric chemistry models using thermal rate coefficients, *k*(*T*,*p*) which may also depend on pressure. It is convenient to parametrize the temperature dependence using modified Arrhenius expressions such as *k*(*T*) = *A*(*T*/298 K)^*n*^ exp(−Δ*E*/*RT*). Here, Δ*E* may include contributions from a positive activation energy or a negative energy of association of a prereaction complex, with these energies defined relative to the reactants.

**Figure 7:**
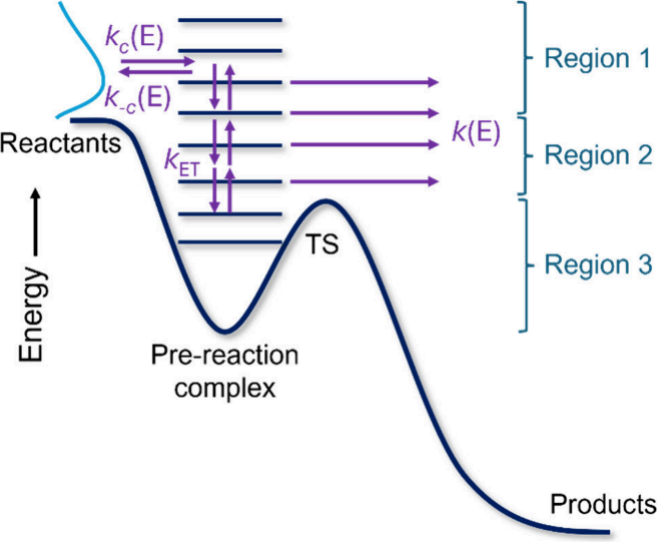
Schematic representation of the energy-grained master equation approach to calculate pressure and temperature dependent rate coefficients. In this example, the dark blue curve shows the potential energy along the reaction coordinate for association of reactants to form a prereaction complex (with forward and reverse rate coefficients *k*_c_ and *k*_–c_), followed by reaction over a submerged transition state (TS). Upward and downward energy transfer in the complex (shown by up and down arrows), with rate coefficients *k*_ET_, is mediated by collisions with a bath gas. Reaction over the transition state is described by energy-dependent rate coefficients *k*(*E*). Three regions can be identified based on the magnitudes of the rate coefficients for these processes. When the molecular complex forms, its initial energy distribution is closely related to that of the thermalized reactants (light blue curve). The complex can be stabilized by collisions to energies lower than those of the reactants, with the microcanonical rate coefficients *k*_c_(*E*) and *k*_–c_(*E*) for complex formation and dissociation becoming zero, while *k*(*E*) for the reactive process remains large for energies above the TS barrier. Collisional events can also trap a fraction of the complex in a third region below the barrier.

Using sufficiently high-level methods of electronic structure theory and large electronic basis sets, modern quantum chemical codes can now compute energies and structures of key species such as complexes, intermediates, and transition states along a bimolecular reaction pathway to “chemical accuracy”, meaning to within about 4 kJ mol^–1^ (often expressed as ∼1 kcal mol^–1^). Values for *k*(*T*,*p*) can then be quantitatively computed using kinetic master equation methods which account for both reaction and (pressure-dependent) collisional energy transfer with a bath gas such as air.^218^ The energy-grained master equation approach involves calculating microcanonical rate coefficients *k*(*E*) for each internal energy grain (see Figure 7 for an illustration) and then solving numerically the coupled differential equations for chemical reaction and energy transfer between grains because of collisions with the bath gas. Master-equation methods are implemented in software packages such as MESMER^219^ and MultiWell.^220^ Important examples discussed at the CECAM workshop include the photochemical oxidation pathways of HFOs, which are planned replacements for hydrofluorocarbons (HFCs) now recognized as significant greenhouse gases. Unintended consequences of HFO use may be significant production of HFC-23 (a long-lived and potent greenhouse gas) and trifluoroacetic acid (TFA), which is a persistent environmental pollutant.^60,221^

### 3.6 Aerosols

Section 2.4 presented an overview of recently developed laboratory approaches to study the absorption of sunlight by atmospheric aerosols and the consequent photochemistry. Explicit simulation of the photochemical behavior of molecular components of nanoscale or larger aerosol droplets lies beyond the capabilities of current computational resources. Nevertheless, theoretical treatments of aspects of photochemistry in aerosols are tractable with available computational methods and judicious approximations. The best available gas-phase pictures of nonadiabatic photochemical pathways often serve as a good starting point for understanding the molecular photodynamics of organic solutes in aqueous solution or other condensed-phase environments, as exemplified by recent studies of UV-excited nitroaromatic molecules which are prevalent in brown carbon aerosols.^222^ The roles of the solvent can then be divided into (i) modifications of the excited-state PESs and the locations of their conical intersections and singlet–triplet crossings by solvent–solute interactions; (ii) vibrational energy transfer to the solvent, which quenches excess internal energy in the organic solute and suppresses some excited-state dynamics; and (iii) provision of new pathways such as excited-state proton transfer (ESPT) to solvent, charge-transfer to solvent, or geminate recombination of radical photoproducts. Some of these changes can be accounted for with approximate continuum dielectric treatments of the solvent environment, but others need inclusion of explicit solvent molecules in the calculations.^223^

The discussion in the CECAM workshop recognized the importance of atmospheric water in photochemical reactions. Computational photochemical simulations must therefore extend to calculations describing photochemistry in molecular complexes and aerosol particles in the atmosphere, whether involving weakly bound dimers with water molecules, molecular nanoclusters, or photochemically active solutes in the micrometer-scale aqueous droplets found in clouds or sea-spray aerosols. Condensed phase chemistry brings new challenges to theory through the (necessarily approximate) treatment of the effects of the surroundings on the molecular photochemistry, but more tractable calculations for organic chromophores microsolvated by a few explicit water molecules in a molecular cluster can serve as useful models to explore bulk solvation effects in an aerosol droplet. As theoretical methods advance, so experimental spectroscopic and mass-spectrometric methods must also evolve to study photochemistry directly in these confined and heterogeneous environments if the effects of physical properties unique to small droplets are to be better understood.^224^

An illustrative example of multiphase chemistry of tropospheric importance is the oxidation of halide ions and release of photoactive halogen-containing molecules such as Cl_2_ from sea salt aerosols, for which a detailed mechanism has recently been unravelled by Gerber and co-workers with the aid of computational calculations and simulations. The uptake of gaseous hypochlorous acid (HOCl) into droplets formed from sea spray and containing dissolved NaCl allows charge transfer from a Cl^–^_(aq)_ ion to the OH in a HOCl–Cl^–^ halogen-bonded complex, with the resulting production of Cl_2_ facilitated by H_3_O^+^ in acidic solutions.^225^ Other similar halide oxidation reactions are possible in aqueous solution, forming species such as ICl that can then photochemically release halogen atoms to drive Cl-atom chemistry in the troposphere. Dissolved organic matter in the sea-surface microlayer can also be incorporated into sea spray aerosols, with certain chromophores absorbing solar radiation and acting as photosensitizers that can induce bulk and interfacial chemistry. Calculations of electronically excited states and the absorption spectra of molecules such as 4-benzoyl benzoic acid (4BBA), which serves as a proxy for more complex organic compounds including humic-like substances, have a valuable role to play in exploring the mechanisms of photosensitization. These calculations can use cluster microsolvation by explicit water molecules to simulate the effects of an aqueous aerosol bulk or interface region, and can examine how the absorption spectrum changes as the pH evolves from pH 7.8 for the sea surface to pH 2–4 for typical aerosols generated from sea spray.^226^

Molecules located at the water–air interface of aqueous aerosol droplets may show photochemistry that is modified from that in the gas phase, as exemplified by calculations from Francisco and co-workers for hydrogen peroxide (H_2_O_2_) on a water droplet surface.^227^ Because the H_2_O_2_ adopts a different geometry at the interface than in the bulk solution or the gas phase, its UV absorption band shifts to longer wavelength and better overlaps the solar spectrum, which accelerates its photolysis by tropospheric solar radiation to produce OH radicals. Further calculations are needed to explore whether this recently reported phenomenon is more generally applicable to the photochemistry of VOCs, many of which preferentially partition to the surfaces of water droplets.

### 3.7 Future Directions in Computational Atmospheric Photochemistry

Over the last decades, atmospheric chemistry research has stimulated the development of new theoretical methods to investigate complex *ground-state* chemical reactions and their rates and mechanisms. Any such connections to theoretical studies of *molecular photochemistry* involving electronically excited states are weaker, despite the importance of photochemistry for current atmospheric models and a strong push from the experimental side to obtain reliable photochemical data for modeling the composition of the atmosphere. Having discussed the current capabilities and limitations of theoretical and computational photochemistry methods in the preceding sections, we can address the question of whether these theories and tools of computational chemistry are now sufficiently mature and quantitatively predictive to provide photochemical and reaction kinetic data of the quality needed for inclusion in computational models of atmospheric chemistry. When such a point is reached, computational calculations of absorption spectra, quantum yields, product branching ratios, and rate coefficients will supplement experimental data accumulated over many years of laboratory measurements. In our opinion, the current answer to this question is nuanced but our outlook is optimistic. Considerable strides have been made in the accurate calculation of absorption spectra (albeit with some caveats), as described in Section 3.3, and bimolecular rate coefficients, as outlined in Section 3.5. Uncertainties in calculated values deduced from efficient quantum chemistry methods can also be quantified by comparing excited-state or reaction-barrier energies with values obtained from more computationally expensive high-level, multireference quantum mechanical calculations.

However, challenges remain to compute properties such as quantum yields and product branching ratios, which require nonadiabatic dynamics simulations. As was discussed in Section 3.2, numerous methods have been devised to treat these dynamics with different degrees of rigor, such as MCTDH, AIMS, and TSH. While the methods to perform the nuclear dynamics and calculate the required electronic-structure quantities have become highly sophisticated, describing the very first step of the dynamics–the photoexcitation process and the resulting initial molecular state of the photoexcited molecule–requires more attention.^146^ In particular, the definition of time scales must be considered,^228^ given the difference between excitation by absorption of incoherent sunlight and by a very short (coherent) laser pulse. The latter type of photoabsorption is what justifies the assumption of sudden excitation generally used in nonadiabatic dynamics.

The computational costs of propagating nuclear wavepackets or classical trajectories prohibit the simulation of long-time (nanosecond or longer) excited-state dynamics associated with some VOCs. Such long-time nonadiabatic dynamics simulations may challenge numerous methods^229^ and reveal issues related to zero-point energy leakage for trajectory-based methods.^230^ However, recent developments within the multiple-spawning framework^231−234^ can reduce the computational burden associated with long-time AIMS simulations. Long time scale processes may be reached by using master equation strategies extended to the nonadiabatic regime, possibly with parametrizations based on quantitative (all-atomistic) TSH trajectories.^217^ For many carbonyl containing VOCs, careful consideration must be given to accurate calculation of spin–orbit couplings so that intersystem crossing pathways can be correctly simulated on picosecond to nanosecond time scales. At atmospheric pressure, collisions with N_2_ or O_2_ will also influence the properties of excited states with nanosecond or longer lifetimes.

A significant challenge remains in the accuracy of electronic-structure theory, in particular, when the balance between different photoproducts may be sensitive to the precise heights of energy barriers or the location of an intersection seam. In addition, a proper description is needed of the internal energy of the system after photoexcitation via the choice of initial conditions. Recent work stressed that the choice of the electronic-structure theory impacts more the results of nonadiabatic molecular dynamics than the strategy employed for the nonadiabatic dynamics.^148^ Other works focusing on the impact of the electronic structure in nonadiabatic dynamics,^235−237^ as well as the various results obtained during an ongoing prediction challenge for the gas-phase photochemistry of cyclobutanone,^238^ further corroborate the central impact of electronic structure in nonadiabatic molecular dynamics.

Perhaps the biggest technical challenge to address in computational atmospheric photochemistry is the accurate treatment of the effects of an aqueous environment or an air–water interface on photochemistry. Growing recognition of the importance of this photochemistry in atmospheric aerosols and clouds is a driver to develop new and accurate methods because of the shortcomings of existing polarizable continuum models and the need to consider explicit water molecules in quantum chemistry and dynamical calculations, potentially in large numbers, to capture correctly the effects of the aqueous environment. Recent developments in polarizable embedding QM/MM strategies,^239,240^ force fields in quantum dynamics,^241^ projector-embedding electronic-structure methods for excited states,^242,243^ and the development of GPU-accelerated electronic-structure methods^244^ constitute examples of strategies that may tackle aqueous atmospheric photochemistry. References (223 and 245) provide additional information about the inclusion of solvent effects in nonadiabatic molecular dynamics.

The theoretical and computational chemistry approaches outlined in Sections 3.1–3.3 provide a roadmap for first-principles calculation of key atmospheric photochemistry processes. Computational methodologies for each of these areas are continually being improved and validated, and some are being built into user-friendly software packages such as AtmoSpec,^195^ but others still require specialist expertise to implement successfully. These methods are already invaluable for interpretation of laboratory measurements of photochemical dynamics using methods such as transient absorption spectroscopy or ultrafast X-ray and electron diffraction. New computational methods are constantly being added to this toolbox, such as the prediction of X-ray absorption and photoelectron spectroscopy, to match advances in experimental capability. If theoretical methods can develop to the point that they directly simulate raw laboratory measurement data such as transient absorption spectra, then some of the challenges of analyzing and interpreting the experimental data might be circumvented.

## 4 From Computational Chemistry to Atmospheric Modeling

As the preceding sections illustrate, theoretical and computational chemistry treatments of absorption spectra and photochemical pathways are providing quantitative results that compare increasingly favorably with the best available experimental data. Computational chemistry is also beginning to fill gaps in the experimental databases, for example, by predicting photochemical properties of molecules that are not readily amenable to experimental study. The question therefore arises of how these computational results can be best used to improve current understanding of the chemistry of the Earth’s atmosphere. Drawing from the best practice developed for translation of laboratory experimental data, the most effective use of new results from computational chemistry is by their incorporation into the chemical schemes used in global, regional, and local models of atmospheric chemistry.^2,20^ The computational results can supplement existing experimental data by filling gaps, serve as validation of some experimental results, replace values of parameters currently included in models as estimates or educated guesses, guide the development of SARs, and expand the range of photochemical processes included in the existing models.^26,27^

An ideal scenario would be comprehensive chemical models that include fully speciated photochemical data, including the pressure, temperature, and–where appropriate–wavelength dependence of absorption cross-sections, quantum yields, product branching ratios, and kinetic parameters. With current restrictions on high-performance computing resources and the human time needed to incorporate the full complexity of such data sets into existing models, this ideal scenario remains impractical to implement in computer simulations of atmospheric chemistry and climate. Instead, parametrizations of the *p*, *T*, and λ dependencies might be sought from new structure activity relationships or use of machine learning approaches trained on computational chemistry data sets.

To promote computational chemistry as a powerful tool for atmospheric chemistry research, there is a clear need to facilitate the uptake of computational photochemistry methods and data by the community of atmospheric chemistry modelers. One way to achieve this might be a one-stop website with user-friendly interfaces for integrated quantum chemistry codes that can compute absorption spectra and photochemical pathways for a molecule of interest. Another could be software that can reliably simulate photochemical reactions in a gaseous mixture, drawing inspiration from computational reactors for ground-state chemistry such as the ab initio Nanoreactor of Martínez and co-workers^246^ or the automated reaction mechanism generation methods of Zádor and co-workers,^247^ Martínez-Núñez and co-workers,^248^ and the GECKO-A^249,250^ and SAPRC-22^26^ atmospheric chemical models. Progress in automated discovery of photochemical reactions is less developed, but efforts are exemplified by the recently reported nonadiabatic Nanoreactor.^251^

Toward such goals, the computational chemistry community is already developing tools with interfaces that enable nonexpert users to generate reliable computational chemistry results. One example is the AtmoSpec code being developed by Hollas et al.,^195^ which has a convenient web interface but carries out high-level electronic structure calculations behind the scenes, using the NEA methods discussed in Section 3 to provide reliable predictions of wavelength-dependent absorption cross-sections (i.e., quantitative calculations of absorption spectra) for the user’s chosen molecule. The availability of such tools and the reliability of their computational predictions need to be effectively communicated to the atmospheric chemistry modeling community. This communication could come through direct collaborations, the type of website proposed above that is either a front-end for or sign-posts the available computational tools, and dissemination at major environmental research conferences. Some of the CECAM workshop participants already work closely with atmospheric chemistry modelers, but a future CECAM workshop bringing together computational photochemists and representatives of the atmospheric chemistry modeling community would also promote stronger links.

## 5 Conclusions

Research in atmospheric chemistry has many environmental and societal benefits. For example, it has identified the impacts of human activity on climate and on stratospheric ozone depletion.^1−3^ In the latter case, and informed by the scientific research, rapid intergovernmental action led to restrictions and then subsequent bans on the use of chlorofluorocarbons (CFCs) and other halocarbons via *the Montreal Protocol on Substances That Deplete the Ozone Layer* and several later revisions. A combination of laboratory studies, field measurements, and computational modeling has also identified HFCs as potent greenhouse gases with high global warming potentials (GWPs). This research led to the Kigali Amendment to the Montreal Protocol to phase out the use of HFCs in applications such as refrigeration, for which HFOs are now proposed as next-generation replacements. Current research, some of which featured in the March 2024 CECAM workshop on *Theoretical and Experimental Advances in Atmospheric Photochemistry*, is now exploring potential long-term risks of the widespread use of HFOs such as secondary production of HFCs and TFA from HFO oxidation chemistry.

Other topical areas of societal importance where computational atmospheric photochemistry can make valuable contributions include air quality, both outside and in indoor environments, where many people spend most of their time. Poor air quality is now a common problem in urban areas worldwide and is recognized to cause numerous health problems. Elevated NO_*x*_ levels, the photochemical production of ozone, and the growth of organic aerosol particles which can be inhaled into the lungs are signatures of poor air quality, and they are processes that can be better understood with input from experts in photochemistry such as those participating in the CECAM workshop.

This Perspective surveys the current frontiers in experimental, theoretical, and computational photochemistry research for molecules of environmental importance, whether in the gas phase or in aqueous aerosol droplets. Its purpose, and that of the CECAM workshop that inspired it, is to explore whether cutting-edge theoretical and computational photochemistry methods can reliably supplement experimental data needed for inclusion in computer models of atmospheric chemistry: in other words, computational (photo)chemistry can bring a new supportive leg to the atmospheric chemistry three-legged stool analogy proposed by Abbatt and co-workers.^21,224^ These data include absorption spectra, photochemical quantum yields, identification of product channels, and reaction rate coefficients for molecules such as VOCs and reactive intermediates with a wide range of atmospheric sources. Although these species are present in the atmosphere only at trace levels that are typically ppb by volume or lower, they contribute significantly to its chemistry and composition. In addition, computational photochemistry simulations provide invaluable mechanistic insights, for example, to guide the development of structure–activity relationships based on physical and chemical principles. Computational chemistry methods can complement laboratory measurements by providing data for molecules, reactive intermediates, and molecular clusters that are challenging to prepare and study in the laboratory. Nevertheless, the computational methods need to be rigorously benchmarked against the best available experimental data to prove their reliability and worth.

A central conclusion from the CECAM workshop is that in some areas, such as calculation of absorption spectra of VOCs, computational methods are now sufficiently mature to provide useful data to atmospheric chemistry modelers, whereas further progress toward the reliable and routine calculation of photochemical quantum yields and product channel branching ratios is still needed. With the development of robust and efficient computational chemistry tools based on rigorous theoretical methods, a higher throughput of calculations should lead to large data sets that can be used to develop SARs or serve as training for machine learning tools, with which the complex chemistry of atmospheric VOCs can be condensed into a form appropriate for incorporation into computer models.

Although the focus of this Perspective is on photochemistry in Earth’s atmosphere, the concepts and methods described are equally applicable to photochemical processes occurring in the atmospheres of other planets and moons in our solar system and in exoplanetary atmospheres. Both these frontiers of atmospheric chemistry are flourishing fields of research, particularly with the growing availability of high-quality observational data, for example, from missions such as Cassini–Huygens and the James Webb Space Telescope (JWST). As an illustration, we see opportunities for computational chemistry to predict how molecular absorption spectra and photochemical reactions change at the high temperatures found in the atmosphere of Venus or in exoplanets such as those classified as Hot Jupiters that are in orbits close to their parent star and perhaps also tidally locked.

There is now a clear need for theoretical and computational photochemists to engage actively with the atmospheric chemistry modeling community, following in the footsteps of the experimental research groups that supply data from laboratory measurements for use in atmospheric models. This dialogue should be two-way, so that the modelers can advise the photochemists on the highest priority questions seeking resolution, for example, by identifying where major discrepancies exist between current model predictions and field measurements of atmospheric composition. Discussion is also needed about how the data from photochemical calculations should be presented to facilitate inclusion in atmospheric models. The sign-posting of new resources such as AtmoSpec should allow modelers to generate their own computational data sets for absorption spectra (and, in the future, other photochemical parameters) to plug gaps identified in their chemical schemes. While such calculations require access to expensive high-performance computing resources, we note that the alternative laboratory measurements also need specialist, often custom-built, equipment that is expensive to assemble, maintain, and operate.

During the CECAM workshop, the participating scientists, all of whom contribute to atmospheric photochemistry research, were challenged to consider whether this research community currently trusts their calculations, simulations, and models sufficiently to propose termination of the use of certain classes of chemicals or of some industrial processes for the wider benefit of humanity and the planetary ecosystem. Precedents include the current global ban on the manufacture and use of CFCs because of the compelling evidence from laboratory and field measurements that their use depletes stratospheric ozone and causes the annual Antarctic ozone hole. Concerns are growing about the environmental consequences of widespread use of fluorinated and perfluorinated organic compounds, some of which are referred to as “forever chemicals”, the accumulation of long-lived and potent greenhouse gases such as SF_6_, and the impacts of choosing HFOs as next-generation refrigerants. The existing evidence for potential harm is not sufficient to advocate a ban on the production and use of HFOs, but scientists such as the CECAM workshop participants have an important role to play in developing any future scientific case that might challenge industry and governments about the real environmental impacts of these and other purportedly benign new chemicals.
